# A Learning-Enhanced Two-Pair Spatiotemporal Reflectance Fusion Model for GF-2 and GF-1 WFV Satellite Data

**DOI:** 10.3390/s20061789

**Published:** 2020-03-24

**Authors:** Yanqin Ge, Yanrong Li, Jinyong Chen, Kang Sun, Dacheng Li, Qijin Han

**Affiliations:** 1 Department of Earth Science and Engineering, Taiyuan University of Technology, Taiyuan 030024, China; geyanqin0064@link.tyut.edu.cn; 2 Lab of Aerospace System and Application, The 54th Research Institute of China Electronics Technology Group Corporation, Shijiazhuang 050081, China; cjygucas@163.com (J.C.); skgucas@163.com (K.S.); 3 Department of Surveying and Mapping, Taiyuan University of Technology, Taiyuan 030024, China; lidacheng@tyut.edu.cn; 4 Department of Remote Sensing Calibration, China Centre for Resources Satellite Data and Application, Beijing 100094, China; hanqj2018@stu.xjtu.edu.cn

**Keywords:** sparse-representation, spatiotemporal fusion, SPSTFM, two image-pair, GF-2 and GF-1 WFV images

## Abstract

Since requirements of related applications for time series remotely-sensed images with high spatial resolution have been hard to be satisfied under current observation conditions of satellite sensors, it is key to reconstruct high-resolution images at specified dates. As an effective data reconstruction technique, spatiotemporal fusion can be used to generate time series land surface parameters with a clear geophysical significance. In this study, an improved fusion model based on the Sparse Representation-Based Spatiotemporal Reflectance Fusion Model (SPSTFM) is developed and assessed with reflectance data from Gaofen-2 Multi-Spectral (GF-2 MS) and Gaofen-1 Wide-Field-View (GF-1 WFV). By introducing a spatially enhanced training method to dictionary training and sparse coding processes, the developed fusion framework is expected to promote the description of high-resolution and low-resolution overcomplete dictionaries. Assessment indices including Average Absolute Deviation (AAD), Root-Mean-Square Error (RMSE), Peak Signal to Noise Ratio (PSNR), Correlation Coefficient (CC), spectral angle mapper (SAM), structure similarity (SSIM) and Erreur Relative Global Adimensionnelle de Synthèse (ERGAS) are then used to test employed fusion methods for a parallel comparison. The experimental results show that more accurate prediction of GF-2 MS reflectance than that from the SPSTFM can be obtained and furthermore comparable with popular two-pair based reflectance fusion models like the Spatial and Temporal Adaptive Fusion Model (STARFM) and the Enhanced-STARFM (ESTARFM).

## 1. Introduction

High Resolution of the Earth Observation System Major Special Project [1] that consists of seven civil satellites covering multispectral, hyperspectral, multi-view and lidar sensors with spatial resolution ranging from 0.8 to 50 m was ratified and started by the State Council, China in May, 2006. Since launched in August 19, 2014, Gaofen-2 (GF-2) A/B satellites carrying a four-channel multispectral camera (spatial resolution is 3.2m) and a panchromatic camera (spatial resolution is 0.8m) aims to be a high-resolution earth observation tool (Table 1). However, the actual annual observation frequency of GF-2 satellites is rather low due to satellite orbital transfer for observation requirements of disasters, emergency events, military, scientific researches, et al, which significantly reduces the application value of GF-2 data especially for available observations. In consideration of similar spectral channels and high observation frequency of four identical Wide-Field-View (WFV-1, WFV-2, WFV-3 and WFV-4) cameras carried by the Gaofen-1 (GF-1) satellite launched in 26 April 2013 [2,3], temporal-spectral information of GF-1 WFV data can be borrowed to GF-2 multispectral images for the spatiotemporal interpolation of GF-2 reflectance data. Among developed spatiotemporal interpolation methods to date, spatiotemporal fusion technique has been proved to be credible owing to its advantages in the synthesis of spatial, temporal and spectral information from multi-source satellite images.

The multi-resource spatiotemporal fusion technique based on surface retrievals like reflectance, temperature, vegetation index and even land cover mapping [4] has been validated as an effective tool to reconstruct time series remotely sensed data with middle-high spatial resolution, and furthermore can be integrated into a spatio-temporal-spectral fusion framework for multisource, multi-view remotely sensed images [5]. Spatiotemporal fusion methods can be classified as different types in accordance with employed mathematic models and their application frameworks [6], or detailed methods in modelling spatiotemporal correlation [7]. Generally, methods based on spectral transformation, unmixing, spatiotemporal smoothing and sparse-learning are mostly developed in current studies. 

Spectral transformation techniques have been introduced to reconstruct a high-resolution multi-spectral image traditionally with unclear temporal information. A notable exception by [8] utilized wavelet transformation to fuse Landsat Thematic Mapper (TM) and Moderate Resolution Imaging Spectroradiometer (MODIS) images, and the resulting image with spatial resolution of 240 m was obtained by replacing the MODIS low-frequency component of the image with the Landsat TM high-frequency component. As another spatiotemporal analysis tool, the unmixing-based fusion model was proposed by [9] and used to estimate high-resolution reflectance from low-resolution values with the least square method, while [10] developed a linear unmixing model. Since only abundance on the class level (the ratio of one-class high-resolution pixels in a low-resolution pixel area) derived from high-resolution data can be obtained, the spatial variability of pixel-level reflectance has not been considered by above two methods. This problem was then addressed by [11] and [12] that introduce spectral information of neighborhood pixels into the unmixing of low-resolution images. For those areas that land cover types changed, methods based on spline interpolation [13] can be introduced to address this problem. 

The Spatial and Temporal Adaptive Fusion Model (STARFM) [14] is considered as the most popular spatiotemporal algorithm and its spatiotemporal adaptation can be improved by reducing errors of different land covers from satellite sensors [15]. Up to now, the STARFM has been widely applied in winter wheat yield estimation [16], evapotranspiration mapping [17], disturbance monitoring [18,19,20], gross primary productivity evaluation [21], classification improvement [22,23], public health studies [24], etc. As to the situation that significant changes of temporal reflectance happened over land covers, [25] developed an Enhanced STARFM (ESTARFM) algorithm applied for a complex heterogeneous land surface. When seasonal characteristics are similar between observation dates and the modelled date, it has higher fusion accuracy, especially for changing land covers like vegetation, and can be improved as a customized fusion model [26]. However, their performances intend to be barely satisfactory while an abrupt land cover change happened [27]. 

A semi-physical spatiotemporal fusion model, in which a backup MODIS reflectance calculation algorithm was separately applied to Landsat and MODIS pixel-scale reflectance, was proposed for addressing the problem caused by the Bidirectional Reflectance Distribution Function (BRDF) [28]. To overcome the scale difference problem in the fusion process, an optimized semi-physical fusion model was developed to accurately predict reflectance changes happened in the scale between high-resolution pixel and low-resolution pixel, and then applied in a regional fusion demonstration [29]. By orderly compositing Regression Model fitting (RM fitting), Spatial Filtering (SF), and Residual Compensation (RC), a spatiotemporal fusion method named Fit-FC was designed to fuse one-pair or two pairs of Sentinel-2 and Sentinel-3 images for generating nearly daily Sentinel-2 images [30]. 

The Sparse Representation-Based Spatiotemporal Reflectance Fusion Model (SPSTFM) is firstly introduced for fusing two observed image pairs (Landsat and MODIS) [31] and then developed with single image pair [32] for a wide application extension. From the view of computation complex and performance for large image patches, an Extreme Learning Machine (ELM) with rich local structural information is introduced to model learning-based spatiotemporal fusion by learning a mapping function on difference images that is also adopted in SPSTFM [33]. Training and learning steps are known to be key for learning-based fusion methods. For instance, dictionary learning step is both employed in two-image-pair-based fusion model and single-image-pair-based fusion model, of which their fusion strategies and detailed steps are strikingly different. For the single-pair learning-based fusion model that combined dictionary learning and high-pass modulation in a two-layer fusion framework, a dictionary training enhanced strategy with spatially or temporally extended training samples was proposed by preliminarily testing Landsat and MODIS multispectral images [34]. 

In this paper, an improved learning-enhanced fusion model is developed by introducing the strategy of spatially extending dictionary training samples to the SPSTFM fusion framework, which is primarily based on weighted difference images reconstruction with dictionary learning. We introduced the details of this improved fusion model in Section 2. Experimental satellite data with similar spectral response function (GF-2 MS and GF-1 WFV) and their fusion results were exhibited in Section 3 and then discussed in Section 4. This paper is finally concluded in Section 5.

## 2. Methods

In this study, the improved model based on the SPSTFM adopted a learning-enhanced strategy to perform the dictionary training process. In detail, inputting two-pair high-resolution images (GF-2 MS) and low-resolution images (GF-1 WFV) at observed dates were spatially-extended for a larger image size than the original inputting image size, and then taken as enhanced training samples into dictionary training and sparse coding steps. By this way, more “overcomplete” high-resolution dictionary and low-resolution dictionary, than those in the SPSTFM can be retrieved in the sparse learning step, and expected to promote the reconstruction accuracy of high-resolution and low-resolution images used in the fusion process. 

The original sparse-learning fusion algorithm SPSTFM consists of three processing steps: (1) Dictionary learning for High-Resolution Difference Image (HRDI) and Low-Resolution Difference Image (LRDI), (2) HRDI reconstruction, and (3) High-Resolution Surface Reflectance (HRSR) reconstruction. Since the high-resolution dictionary $D_{h}$ of HRDI and the low-resolution dictionary $D_{l}$ of LRDI are both retrieved from dictionary learning operation, the completeness of $D_{h}$ and $D_{l}$ therefore significantly affects the accuracy of presentation and prediction of high-resolution image at modeled date. 

**Figure 1:**
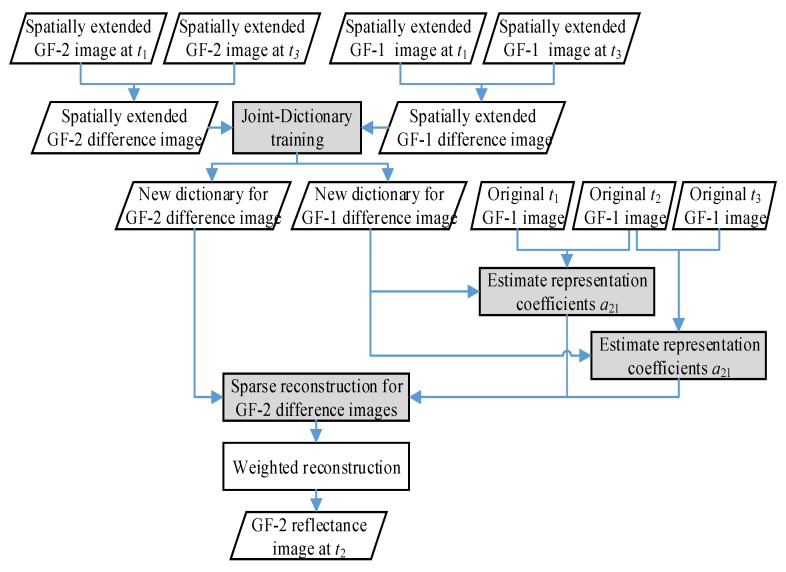
Flow chart of the proposed fusion method in this paper.

In this study, an improved sparse-learning scheme for two image pair fusion was developed by promoting the accuracy of dictionary learning process. The main idea of the proposed fusion scheme was to perform the dictionary training process by using spatially-extended training samples with larger space range than original input training images. In this way, newly retrieved high-resolution and low-resolution overcomplete dictionaries $D_{h}^{\prime }$ and $D_{l}^{\prime }$, with higher completeness than those derived from the dictionary training operation with original training samples, can be obtained. The flow chart of the proposed fusion scheme is shown in Figure 1. 

At the beginning of the proposed fusion scheme, spatially-extended high-resolution images ($H_{1}^{\prime }$ and $H_{3}^{\prime }$) and corresponding low-resolution images ($L_{1}^{\prime }$ and $L_{3}^{\prime }$) with the same image size at two observed dates t1 and t3 were collected and then utilized to generate inputting training samples for subsequent dictionary learning process. Note that the pattern of the spatial extension of above-mentioned high-resolution images and low-resolution images is performed by extending each image boundary (upper, lower, left and right) with the same size. In this way, similar types of surface features with spectrally similar characteristics can be expected in neighborhood pixels of original images. When the spatially-extended size of $H_{1}^{\prime }$, $L_{1}^{\prime }$ and $H_{3}^{\prime }$, $L_{3}^{\prime }$ was determined, a new high-resolution difference image $H_{13}^{\prime }=H_{1}^{\prime }-H_{3}^{\prime }$ and a new low-resolution difference image $L_{13}^{\prime }=L_{1}^{\prime }-L_{3}^{\prime }$ were calculated and then taken as updated training samples. Thus, new high-resolution and low-resolution overcomplete dictionaries $D_{h}^{\prime }$ and $D_{l}^{\prime }$ can be retrieved by optimizing the formula expressed below:

(1) $$\left\{D_{h}^{\prime },D_{l}^{\prime },\alpha_{13}^{\prime }\right\}=\underset{D_{h}^{\prime },D_{l}^{\prime },\alpha_{13}^{\prime }}{\arg\min}\left\{\|H_{13}^{\prime }-D_{h}^{\prime }\alpha_{13}^{\prime }\|{}_{2}^{2}+\|L_{13}^{\prime }-D_{l}^{\prime }\alpha_{13}^{\prime }\|{}_{2}^{2}+\lambda \|\alpha_{13}^{\prime }\|{}_{1}\right\}$$

where $\alpha_{13}^{\prime }$ is the corresponding sparse coefficient for both new high-resolution difference image $H_{13}^{\prime }$ and new low-resolution difference image $L_{13}^{\prime }$, λ is the Lagrange multiplier. By introducing a joint sparse coding method named K-Means Singular Value Decomposition (K-SVD) based on coupled dictionary training, the formula (1) can be transformed as:

(2) $$\left\{D_{joint}^{\prime },\alpha_{13}^{\prime }\right\}=\underset{D_{joint}^{\prime },\alpha_{13}^{\prime }}{\arg\min}\left\{\|Z^{\prime }-D_{joint}^{\prime }\alpha_{13}^{\prime }\|{}_{2}^{2}+\lambda \|\alpha_{13}^{\prime }\|{}_{1}\right\}$$

where $D_{joint}^{\prime }=\left[D_{h}^{\prime },D_{l}^{\prime }\right]$, $Z^{\prime }=\left[H_{13}^{\prime },L_{13}^{\prime }\right]$. Here, the original SPSTFM algorithm uses image blocking strategy to train high-resolution and low-resolution difference images, and the default image patch size is set as 7 × 7 pixels, the number of atoms is set as 2000 for training a difference image with 500 × 500 pixels. In consideration of spatial extension of new high-resolution difference image and low-resolution difference image utilized for dictionary training, the number of atoms for the new training image size should be reassigned a higher value (above 2000) with the same patch size (7 × 7 pixels). 

Once $D_{h}^{\prime }$ and $D_{l}^{\prime }$, respectively, for new high-resolution and low-resolution difference images were retrieved, updated sparse coefficient $\alpha_{21}^{k^{\prime }}$ for the $k^{\prime }$-th difference image patch between t1 and t2 can be expressed by the corresponding $k^{\prime }$-th low-resolution difference image patch $x_{21}^{k^{\prime }}$ and the new dictionary $D_{l}^{\prime }$:

(3) $$\alpha_{21}^{k^{\prime }}=\underset{\alpha_{21}^{k^{\prime }}}{\arg\;\min}\frac{1}{2}\|x_{21}^{k^{\prime }}-D_{l}^{\prime }\alpha_{21}^{k^{\prime }}\|{}_{2}^{2}+\lambda \|\alpha_{21}^{k^{\prime }}\|{}_{1}$$

The $k^{\prime }$-th HRDI patch $y_{21}^{k^{\prime }}$ between t1 and t2; therefore, can be solved by:

(4) $$y_{21}^{k^{\prime }}=D_{h}^{\prime }\alpha_{21}^{k^{\prime }}$$

The $k^{\prime }$-th HRDI patch $y_{32}^{k^{\prime }}$ between t2 and t3 can be calculated in the same way as $y_{21}^{k^{\prime }}$. On a basis of predefined weighting parameters $\omega_{1}^{k^{\prime }}$ and $\omega_{3}^{k^{\prime }}$, the $k^{\prime }$-th high-resolution surface reflectance (HRSR) image patch at the modeled date t2 can finally be predicted as:

(5) $$H_{2}^{k^{\prime }}=\omega_{1}^{k^{\prime }}\times \left(H_{1}^{k^{\prime }}+y_{21}^{k^{\prime }}\right)+\omega_{3}^{k^{\prime }}\times \left(H_{3}^{k^{\prime }}+y_{32}^{k^{\prime }}\right)$$

where $H_{1}^{k^{\prime }}$, $H_{3}^{k^{\prime }}$ are the $k^{\prime }$-th high-resolution surface reflectance (HRSR) image patch, respectively, at the observed date t1 and t3. The whole HRSR image at the modelled date t2 is derived by mosaicking each HRSR image patch $H_{2}^{k^{\prime }}$.

In consideration of the heterogeneity and the diversities of the spatial extension of surface features in different directions, a robust extension strategy was to drive spatially-extended directions of training samples to yield to the same central position from their original fusion images. However in our preliminary experiment, fusion quality of the proposed method was not so sensitive to the spatial extension directions. In this study, GF-2 and GF-1 WFV reflectance images acquired at two observed dates were cropped as different image sizes to validate the effect of training-sample sizes on fusion quality of proposed method. A spatially-extended areas with the actual study area as the center and covering 64 km^2^ (2000 × 2000 GF-2 pixels) was determined as the maximum image size of training samples just inputted in the sparse coding process. Moreover, 20 groups of training samples with image size ranging from 500 × 500 GF-2 pixels to 2000 × 2000 GF-2 pixels were gradually obtained with a resized step of 0.4 × 0.4 km (100 × 100 GF-2 pixels). As a result, spatially-extended study areas covered by two-pair high-resolution and low-resolution images at observed dates were orderly classified to training samples with image sizes resized as 500 × 500, 600 × 600, …, 2000 × 2000 GF-2 pixels. Note that, above spatially-extended training samples were only used in the dictionary learning process rather than the weighting calculation for predicting GF-2 reflectance at the modelled date.

## 3. Results

### 3.1. Study Area and Data Preprocessing

**Figure 2:**
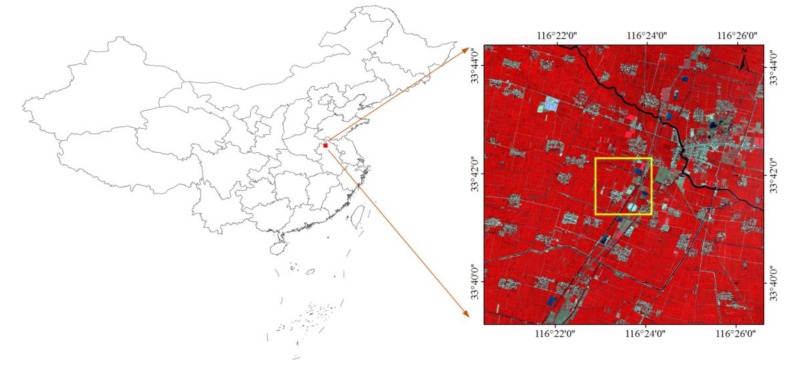
The selected study area in this study and the corresponding Gaofen-2 (GF-2) false-color composite of NIR (Near-Infrared), red and green bands acquired on 30 April 2017.

To avoid the effect of shadows from buildings and mountains on the fusion between high-resolution images and low-resolution images, a study area located in the North China Plain (Shandong province, China) covering cropland, residential area (low rise buildings), and water body was selected to perform fusion experiments. As shown in Figure 2, only the center part of the study area covering 2 × 2 km (500 × 500 GF-2 pixels) takes part in fusion process (see yellow solid box in Figure 2), and employed satellite images with larger image size than the center part are taken as training samples for the proposed sparse learning-based fusion scheme. 

**Figure 3:**
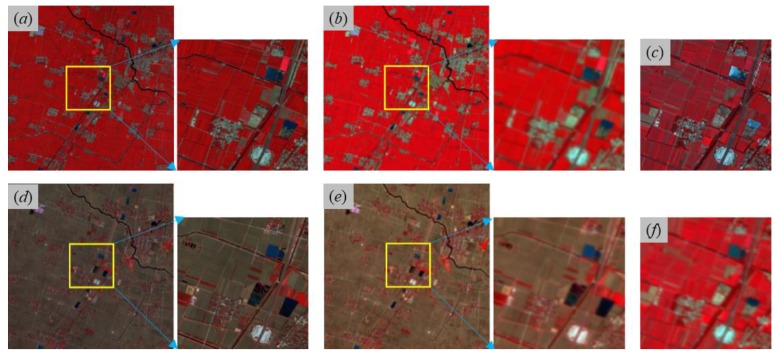
Employed GF-2 and GF-1 WFV images in this study. (**a,d**) and (**b,e**) are, respectively, GF-2 images acquired on 30 April, 8 November 2017 and GF-1 WFV images acquired on 29 April 8 November 2017 with 2000 × 2000 GF-2 pixels (6.4 km^2^). The center image of (**a,b,d,e**) covers 500 × 500 GF-2 pixels (yellow solid box) and is then used in all fusion experiments companying with (**f**) GF-1 WFV image acquired on July 24, 2017. (**c**) is the actual GF-2 image acquired on 23 July 2017.

**Figure 4:**
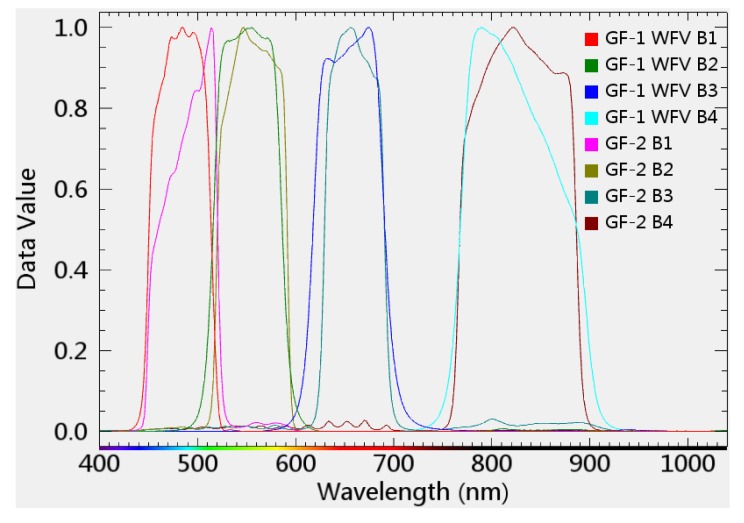
Spectral response curves of multispectral channels (blue, green, red, and NIR) from the GF-2 sensor and GF-1 WFV multispectral sensors.

GF-2 multispectral images acquired on 30 April, 23 July, and 8 November 2017 and corresponding GF-1 WFV images acquired on 29 April, 24 July and 8 November 2017, respectively, shown in Figure 3a,c,d,b,f,e are collected and then employed for substantial fusion experiments, in which the GF-2 image acquired on 23 July 2017 is used for the validation of fusion results and other images from GF-2 and GF-1 WFV are used to perform fusion methods in this study. Since GF-2 and GF-1 WFV data have very similar spectral channels, band width and spectral response function, no extra spectral normalization operation for GF-2 and GF-1 WFV images are needed before fusion. For a reliable reflectance computation, all above experimental images with DN (Digital Number) values are radiometrically corrected, and converted to surface reflectance by atmospheric correction and then rescaled to [0, 10,000]. Since spectral response characteristics of the Gaofen-2 multispectral sensor is exactly similar with Gaofen-1 WFV satellite sensors (Figure 4), their land surface reflectance data produced from atmosphere correction have no need of radiometric normalization. After geometrical correction, GF-2 images are up-resampled from 3.2 to 4 m for a receivable spatial scale, and GF-1 WFV images are accordingly down-resampled from 16 to 4 m for pixel-to-pixel fusion processing, and then GF-1 WFV images are registered to GF-2 images. 

### 3.2. Experimental Results

To give a credible description on fusion quality, seven quantitative indices (Table 2) including Average Absolute Deviation (AAD), Root-Mean-Square Error (RMSE), Peak Signal to Noise Ratio (PSNR), Correlation Coefficient (CC), Spectral Angle Mapper (SAM) [35], Structure Similarity (SSIM) [36] and Erreur Relative Global Adimensionnelle de Synthèse (ERGAS) [37] are chosen to validate fusion results from employed fusion methods.

(6) $$SAM=\cos^{-1}\left(\frac{\sum_{i=1}^{B}\rho_{P_{i}}\rho_{R_{i}}}{\sqrt{\sum_{i=1}^{B}\rho_{P_{i}}^{2}}\sqrt{\sum_{i=1}^{B}\rho_{R_{i}}^{2}}}\right)$$

(7) $$SSIM_{i}=\frac{\left(2\mu_{P_{i}}\mu_{R_{i}}+C_{1}\right)\left(2\sigma_{P_{i}R_{i}}+C_{2}\right)}{\left(\mu_{P_{i}}^{2}+\mu_{R_{i}}^{2}+C_{1}\right)\left(\sigma_{P_{i}}^{2}+\sigma_{R_{i}}^{2}+C_{2}\right)}$$

(8) $$ERGAS=100\frac{p}{r}\sqrt{\frac{\sum_{i=1}^{B}{\left(RMSE_{i}\right)}^{2}}{B}}$$

where $\rho_{P_{i}}$ and $\rho_{R_{i}}$ indicate reflectance in band i∈[1,B] of modeled image P and actual image R; $\left(\mu_{P_{i}},\mu_{R_{i}}\right)$, $\left(\sigma_{P_{i}},\;\sigma_{R_{i}}\right)$, and $\sigma_{P_{i}R_{i}}$ correspond to the mean value, standard deviation, and covariance in band i of P and R, respectively; $C_{1}={\left(k_{1}*L\right)}^{2}$ and $C_{2}={\left(k_{2}*L\right)}^{2}$; $k_{1}$ and $k_{2}$ are generally set as 0.01 and 0.03; L is the grayscale of reflectance images; $RMSE_{i}$ is the RMSE in band i of P and R; p and r are the spatial resolutions of P and R. Small values of RMSE, SAM, and ERGAS and a high value of SSIM between modeled reflectance and actual reflectance indicate a high fusion quality. 

Band-based scatter plots are provided to analyze agreements between predicted GF-2 reflectance and actual GF-2 reflectance and elapsed time is additionally recorded to evaluate the efficiency of fusion procedures. As a parallel comparison, four spatiotemporal fusion models based on two-pair observed high-resolution images and low-resolution images (SPSTFM, the proposed method, STARFM and ESTARFM) are employed to perform experimental data described in Section 3.1. Note that only images in yellow solid boxes of Figure 3a,b,d–f, all with 500 × 500 pixels are required for inputs of SPSTFM, STARFM, and ESTARFM algorithms, while spatially-extended images with a maximum size 2000 × 2000 pixels shown in the left image of Figure 3a,b,d,e are used as additional auxiliary data for enhanced dictionary training in the proposed method. Moreover, the proposed fusion procedure is programed by calling ksvdbox [38] and ompbox [39] in MATLAB 2014b under Microsoft Windows-7 64-bit system with CPU Intel Core i7 (3.4 GHz) and RAM 16 GB.

In SPSTFM algorithm, an image blocking strategy is adopted in dictionary training process and related parameters are defined primarily as default. For an image with 500 × 500 pixels, default patch size of both high-resolution difference image and low-resolution difference image is chosen as 7 × 7 pixels, default sparsity parameter is taken as 0.1, and the default number of dictionary atoms (size of dictionary codebook) is set as 256. In order to run an essential dictionary training procedure, sufficient training sample patches are therefore required, so that the number of training sample patches in SPSTFM is adjusted as 6000 rather than 2000 used by (Song and Huang 2013). As to the proposed method, training sample sizes from 600 × 600 pixels to 2000 × 2000 pixels (with an interval of 100 × 100 pixels) that cover larger spatial areas than the area used in SPSTFM, are prepared for training high-resolution and low-resolution dictionaries separately with 15 spatially-extended difference images. Default parameters including patch size, sparsity parameter and number of atoms are set to be the same as SPSTFM, while the number of patches num_patch can be calculated according to the assigned training sample size size_ts:

(9) $$num\_patch=\frac{size\_ts^{2}}{500^{2}}\times 6000$$

where 500 and 6000 match the size_ts and num_patch of SPSTFM algorithm. The calculation based on the area ratio between extended training image and original training image provides a fair assignment for SPSTFM and the proposed method. By this way, 8640 and 96,000 of patches are respectively assigned to train difference images with 600 × 600 and 2000 × 2000 pixels. 

In order to optimize fusion quality of STARFM and ESTARFM algorithms, running parameters especially for searching spectrally similar pixels from neighbor pixels are determined as default. As a result, the moving window size is set as three times that of GF-1 WFV pixel that is 48 × 48 m (about 12 × 12 GF-2 pixels). Uncertainty parameter in STARFM for assessing spectral differences between temporal GF-1 WFV images and between corresponding pixels from GF-2 image and GF-1 WFV image is defined as 50 (0.5% of the maximum of stretched reflectance) for both GF-2 and GF-1 WFV data, while its default value in ESTAFM is set as 0.2% of the maximum of stretched reflectance (about 20). The number of land cover types is also important for filtering spectrally similar pixels that are employed to calculate weighting contributions to predicted pixel reflectance. Although this parameter firstly defined in STARFM is utilized in the same way as ESTARFM, our preliminary experiment shows that fusion quality has not been improved but reduced when different levels of adjustments for their default land cover types are made for STARFM and ESTARFM. In this experiment, the number of land cover types respectively are set as 40 classes for STARFM and four classes for ESTARFM. 

**Figure 5:**
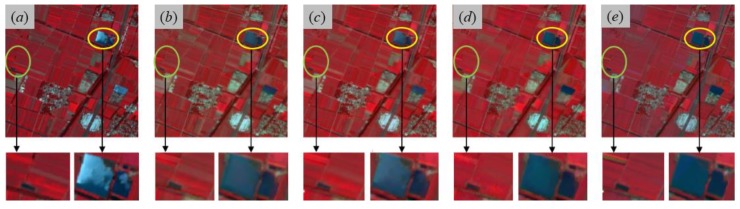
Actual and predicted GF-2 reflectance images (composite of green, red, and NIR bands) at 23 July 2017, respectively, from (**a**) actual reflectance image, (**b**) SPSTFM, (**c**) the proposed method with the training sample size as 2000 × 2000 pixels, (**d**) STARFM, and (**e**) ESTARFM algorithms. Image areas covered by green and yellow ovals indicate farmland and water body that are both zoomed in below the corresponding full image.

**Figure 6:**
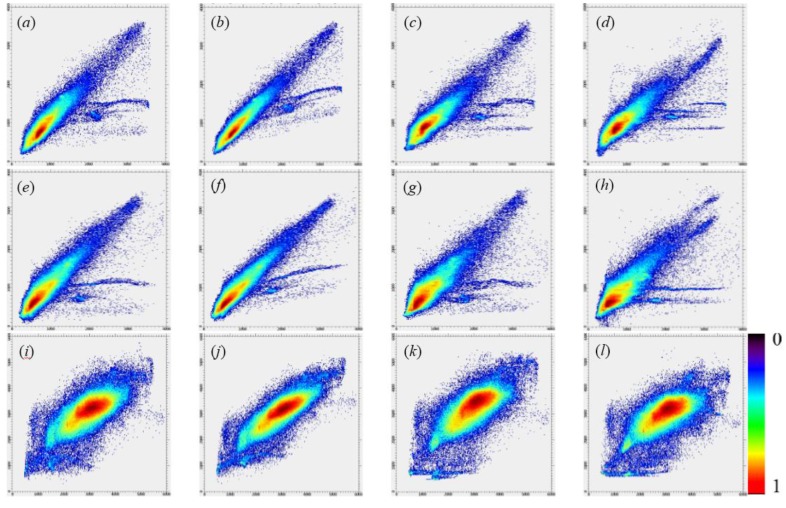
Channel-based scatter plots between actual reflectance (X-axis) and predicted reflectance (Y-axis) from employed fusion algorithms. (**a**–**d**), (**e**–**h**), and (**i**–**l**) are, respectively, green, red, and NIR reflectance predicted by SPSTFM, the proposed method, STARFM and ESTARFM. The numbers 0 and 1 in the top and bottom of the density slice legend indicate a sparse and a dense plot distribution.

**Figure 7:**
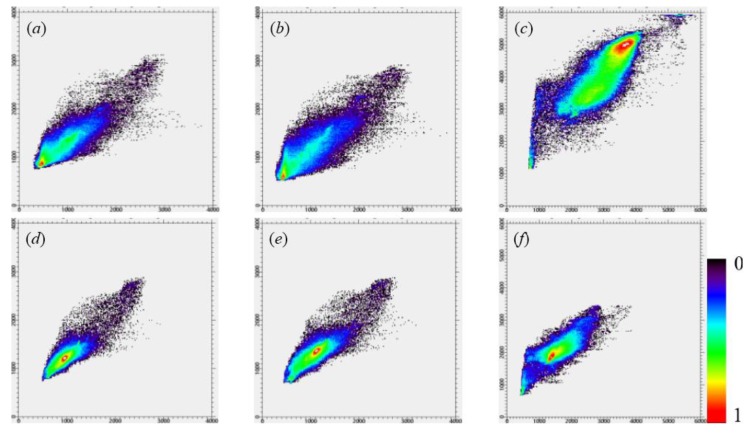
Scatter plots of GF-2 reflectance and GF-1 WFV reflectance acquired on 29 April and 8 November 2017. (**a**–**c**) and (**d**–**f**) separately show reflectance agreements in green, red, and NIR bands for GF-2 (500 × 500 pixels) and GF-1 WFV (500 × 500 pixels) image-pair observed at 29 April and 8 November 2017, respectively.

The predicted GF-2 reflectance images at the modeled date (23 July 2017) from employed fusion models including SPSTFM, the proposed method with the training sample size as 2000 × 2000 pixels, STARFM and ESTARFM are finally shown in Figure 5 with the composite of green, red, and NIR channels. All assessment indices including AAD, PSNR, CC, RMSE, SAM, SSIM indices for each band and ERGAS index for overall bands are listed in Table 2, where the training sample size used in SPSTFM is 500 × 500 pixels and 15 different training sample sizes used in the proposed method are ranging from 600 × 600 to 2000 × 2000 pixels. To find out agreements between predicted GF-2 reflectance and actual GF-2 reflectance, scatter plots are regarded as an additional analysis tool for validating fusion quality on green band, red band, and NIR band (Figure 6). Band-based agreement between GF-2 reflectance and corresponding GF-1 WFV reflectance acquired on 29/30 April and 8 November 2017 are also shown with scatter plots in Figure 7.

## 4. Discussion

Acceptable predicted results from SPSTFM, the proposed method, STARFM and ESTARFM in Figure 5 can be obtained by fusing two-pair-observed GF-2 and GF-1 WFV images. From a visual point of view, results from learning-based methods (SPSTFM and the proposed method) generally have a better color restoration of actual GF-2 composite images than results from STARFM and ESTARFM. For instance, predicted farmland and water body (yellow and green ovals in Figure 5e) in the fused image of ESTARFM show an obvious spectral distortion in comparison with the actual GF-2 composite image. This problem is probably caused by the seasonal discrepancy between three GF-1 reflectance images acquired on 29 April, 24 July and 8 November 2017 (see Figure 3b,e,f), which leads to unstable multiplicative coefficients a in linear models respectively established with GF-2 and GF-1 WFV reflectance acquired on 29 April 2017, and GF-2 and GF-1 WFV reflectance acquired on 8 November 2017. The explanation is well supported by the SSIM index calculated for STARFM and ESTARFM in Table 2 and channel-based scatter plots between Figure 6c,g,k from STARFM and Figure 6d,h,l from ESTARFM. 

In view of spatial information restoration, learning-based methods can provide more spatial texture details than STARFM especially for changing farmland (Figure 5). ESTARFM has a lower ERGAS index and average SAM index than corresponding ERGAS index and average SAM index from STARFM (Table 2). A low performance of SPSTFM in assessment indices can be addressed by the dictionary atoms defined in the dictionary learning and sparse coding process. A significant promotion of fusion quality therefore can be expected by reduce the number of dictionary atoms. 

Moreover, results from the proposed method based on sparse learning give a favorable performance both in avoiding spectral distortion and capturing spatial texture details. The average of AAD, PSNR, CC, RMSE, SAM, SSIM, and the ERGAS index derived from SPSTFM are orderly improved when training sample size are spatially-extended from 500 × 500 to 2000 × 2000 pixels (Table 2). Scatter plots of green, red, and NIR bands shown in Figure 6b,f,j indicate a high agreement between actual GF-2 reflectance and predicted GF-2 reflectance from the proposed method, of which density plots have a more concentrated distribution than that in Figure 6a,e,i from SPSTFM, Figure 6c,g,k from STARFM and Figure 6d,h,l from ESTARFM. An effective improvement for fusion quality of SPSTFM, STARFM, and ESTARFM; therefore, can be expected by the proposed method with training sample size above 1200 × 1200 pixels, while it cannot have a significant growth after training sample size larger than 1500 × 1500 pixels. In general, the completeness of learned dictionary from spatially-extended training samples will not be lower than that from original training samples without spatially extension, which can be attributed to learning mechanism of sparse coding for dictionary training. Considering the fact that spatially-extended image areas have similar land cover types and inner-class heterogeneity with the original training sample image, the completeness of proposed dictionary training strategy would be significantly promoted. While similar land cover types are absent in those spatially-extended image areas, updated atoms in trained dictionaries calculated from extended training image areas probably have low correlation and also slightly promote the completeness of sparse dictionary. 

Green band (Figure 6a,b,c,d) and red band (Figure 6e,f,g,h) generally have higher fusion accuracy than the NIR reflectance (Figure 6i,j,k,l) for all employed fusion algorithms. In Table 2, assessment indices from the green band and red band have a higher AAD, RMSE, SAM and a lower PSNR, CC, SSIM than that from NIR band for all fusion algorithms. The reason may attribute to the spectral correlation of surface features between GF-2 and GF-1 WFV images at two observed dates. Figure 7a,b,c,d,e,f, respectively, show reflectance agreements between green, red, and NIR bands of GF-2 and GF-1 WFV acquired on 29/30 April and 8 November 2017. A rather low agreement on the NIR band of GF-2 reflectance with GF-1 WFV reflectance can be observed, while an acceptable agreement is provided for reflectance of both the green band and the red band.

The executing time of fusion procedures is finally regarded as an important index to assess efficiency of fusion algorithms. In this respect, STARFM just costs about 10 s for blending images with 500 × 500 pixels, while ESTARFM costs 348.56 s (its fast version costs 228. 21 s). As to sparse learning-based fusion procedures, the elapsed time ranges from 60.95 s to 323.44 s with training sample size from 500 × 500 to 2000 × 2000 pixels and the number of patches from 6000 to 96,000. On the other side, the relationship between elapsed time and training sample size of the proposed method intends to be a rapid increasing rather than a linear growth. Hence, a proper size for selecting training samples can balance the efficient and accuracy of the proposed method.

## 5. Conclusions

For the purpose of blending two-pair observed high-resolution images and low-resolution images, an improved sparse-learning fusion method was developed by introducing an existing strategy of spatially extending training samples to the dictionary learning process, and then applied to Gaofen satellite data (GF-2 MS and GF-1 WFV). Employed assessment indices AAD, PSNR, CC, RMSE, SAM, SSIM, and ERGAS were used to evaluate the models’ performance and the conclusions of this study were:

(i) When observed training samples spatially increased, the improved fusion model can promote prediction accuracy of the SPSTFM for blending GF-2 MS and GF-1 WFV reflectance images, and; therefore, can be expected to be effective for multisource remotely sensed data of which the spatial scale difference significantly affected the fusion quality of results from the single-pair-based fusion algorithm.

(ii) Compared to current popular two-pair spatiotemporal fusion models, including STARFM and ESTARFM, a better performance of the improved fusion model can be obtained while a time-consuming problem; therefore, will be generated. Fortunately, this problem can be addressed by the improvement of sparse coding process.

(iii) Inputting data quality and their agreement in spatiotemporal-spectral dimensions are important to results from spatiotemporal fusion methods, including the improved fusion model, which can also lead to the discrepancy between predicting accuracy and spectral channels of GF-2 MS images. In fact, because the GF-2 MS and GF-1 WFV sensors have a high similarity in spectral response function, the radiometric agreement problem can be hardly considered in our fusion experiment.

(iv) Shadows, especially building shadows, usually exists in high-resolution images (GF-2) owing to effects of imaging geometry on urban areas. For fusion models that needs two observed image pairs of GF-2 MS and GF-1 WFV in this paper, building shadows caused from two observed imaging geometric conditions probably lead to a temporal discrepancy in shadow areas. Unfortunately, building shadows is hard to be effectively removed with one observed high-resolution image, which is indeed a challenge problem for the single-pair learning-based fusion model. On the other hand, this problem can be addressed in the framework of two-image-pair-based fusion methods. For instance, overlapped shadow areas in two or more observed high-resolution images can be kept while other types of shadows can be removed using temporal corresponding clear areas, by which a high radiometric agreement between or among temporal high-resolution reflectance data can be expected. 

## Figures and Tables

**Table 1:** Employed Gaofen-2 (GF-2) and Gaofen-1 Wide-Field-View (GF-1 WFV) multispectral data for fusion experiments.

Band Name	GF-2 Multispectral				GF-1 WFV			
	Band Width	Spatial Resolution	Revisit Cycle	Employed Dates	Band Width	Spatial Resolution	Revisit Cycle	Employed Dates
Blue	0.45–0.52 μm	4 m	5 days	04/30/2017 07/23/2017 11/08/2017	0.45–0.52 μm	16 m	2 days	04/29/2017 07/24/2017 11/08/2017
Green	0.52–0.59 μm				0.52–0.59 μm			
Red	0.63–0.69 μm				0.63–0.69 μm			
NIR	0.77–0.89 μm				0.77–0.89 μm			

**Table 2:** Assessment indices Average Absolute Deviation (AAD), Root-Mean-Square Error (RMSE), Peak Signal to Noise Ratio (PSNR), Correlation Coefficient (CC), Spectral Angle Mapper (SAM), Structure Similarity (SSIM) and Erreur Relative Global Adimensionnelle de Synthèse (ERGAS) of fusion quality from different fusion algorithms.

Methods	Training Sample Size	AAD × 10^2^			PSNR			CC			ERGAS
		Green	Red	NIR	Green	Red	NIR	Green	Red	NIR	
SPSTFM	500 × 500	1.67	1.58	4.72	23.9890	23.9886	20.6887	0.8348	0.8206	0.6978	30.2124
Proposed fusion model	600 × 600	1.54	1.57	4.69	23.7903	24.2777	20.9278	0.8391	0.8358	0.7146	28.9806
	700 × 700	1.49	1.55	4.64	23.6544	24.4698	21.3056	0.8445	0.8380	0.7227	27.5489
	800 × 800	1.44	1.52	4.58	23.3901	24.6415	21.5419	0.8489	0.8447	0.7266	28.1647
	900 × 900	1.44	1.50	4.53	23.2203	24.9902	21.6763	0.8533	0.8493	0.7304	26.5306
	1000 × 1000	1.40	1.49	3.49	23.1411	25.1369	21.8535	0.8550	0.8525	0.7341	26.1852
	1100 × 1100	1.37	1.47	3.45	24.0155	25.4554	22.0025	0.8582	0.8566	0.7369	25.9577
	1200 × 1200	1.36	1.46	3.37	24.1223	25.5109	22.3117	0.8597	0.8584	0.7397	24.5226
	1300 × 1300	1.33	1.43	3.30	24.3005	25.6688	22.7006	0.8623	0.8631	0.7434	25.0774
	1400 × 1400	1.28	1.44	3.25	24.4379	25.6979	22.8990	0.8644	0.8679	0.7482	23.4095
	1500 × 1500	1.25	1.41	3.11	24.7706	25.7452	23.1453	0.8679	0.8694	0.7527	23.1710
	1600 × 1600	1.24	1.39	2.81	24.8269	25.7885	23.3366	0.8688	0.8728	0.7573	23.0139
	1700 × 1700	1.22	1.37	2.72	24.9901	25.8503	23.4210	0.8702	0.8755	0.7610	23.8441
	1800 × 1800	1.19	138	2.70	25.1796	25.9116	23.6962	0.8731	0.8771	0.7644	23.2267
	1900 × 1900	1.18	1.36	2.67	25.3661	25.9820	23.8331	0.8756	0.8797	0.7697	22.9553
	2000 × 2000	1.18	1.34	2.66	25.4157	25.9833	23.8400	0.8754	0.8800	0.7711	22.9874
STARFM	—	1.75	1.56	4.38	23.5678	24.1568	20.6543	0.8533	0.8496	0.7301	26.0771
ESTARFM	—	1.69	1.47	3.53	23.4508	24.3378	21.6888	0.8384	0.8517	0.7009	25.9248
**Methods**	**Training Sample size**	**RMSE ×10^2^**			**SAM**			**SSIM × 10^2^**			**Elapsed Time (s)**
		**Green**	**Red**	**NIR**	**Green**	**Red**	**NIR**	**Green**	**Red**	**NIR**	
SPSTFM	500 × 500	2.41	3.36	6.47	1.7829	1.7880	1.8993	87.08	73.61	61.18	60.95
Proposed fusion model	600 × 600	2.29	3.21	6.29	1.7863	1.7563	1.8563	87.94	83.94	67.94	67.59
	700 × 700	2.22	3.17	6.31	1.7855	1.7655	1.8109	88.17	84.57	71.17	88.26
	800 × 800	2.31	2.94	5.86	1.7745	1.7445	1.8245	87.34	85.46	72.46	91.75
	900 × 900	2.15	2.85	5.45	1.7833	1.7492	1.8133	88.26	86.34	73.34	96.30
	1000 × 1000	2.10	2.77	5.38	1.7790	1.7543	1.8046	88.42	87.09	75.07	110.17
	1100 × 1100	2.23	2.63	5.29	1.7811	1.7415	1.7811	89.33	87.98	74.82	122.63
	1200 × 1200	2.19	2.49	5.31	1.7794	1.6893	1.7914	89.92	88.44	74.69	135.76
	1300 × 1300	2.15	2.51	5.15	1.7807	1.6780	1.8087	89.77	88.17	74.57	166.44
	1400 × 1400	2.16	2.36	4.91	1.7765	1.6884	1.7947	89.89	89.29	75.92	179.28
	1500 × 1500	2.01	2.27	4.84	1.7636	1.6731	1.7882	90.18	89.42	76.34	201.16
	1600 × 1600	2.01	2.25	4.86	1.7679	1.7379	1.8009	90.14	89.64	76.64	227.81
	1700 × 1700	2.02	2.31	4.90	1.7685	1.7475	1.7852	90.07	88.83	75.89	250.66
	1800 × 1800	2.01	2.27	4.84	1.7521	1.7551	1.7801	90.10	89.25	76.41	279.05
	1900 × 1900	2.03	2.24	4.91	1.7469	1.7460	1.7767	90.16	89.71	76.16	311.68
	2000 × 2000	2.02	2.22	4.88	1.7514	1.7471	1.7823	90.18	90.06	76.81	323.44
STARFM	—	2.40	2.45	5.50	1.7812	1.7605	1.8181	87.22	87.47	75.19	10.78
ESTARFM	—	2.41	2.48	5.02	1.7749	1.7538	1.8126	85.80	86.36	70.27	348.56
